# Cilia, calcium and the basis of left-right asymmetry

**DOI:** 10.1186/1741-7007-10-102

**Published:** 2012-12-19

**Authors:** Dominic P Norris

**Affiliations:** 1 Mammalian Genetics Unit, MRC Harwell, Harwell Science and Innovation Campus, Oxfordshire, OX11 0RD, UK

## Abstract

The clockwise rotation of cilia in the developing mammalian embryo drives a leftward flow of liquid; this genetically regulated biophysical force specifies left-right asymmetry of the mammalian body. How leftward flow is interpreted and information propagated to other tissues is the subject of debate. Four recent papers have shed fresh light on the possible mechanisms.

## Opinion

Although we can instinctively tell left from right, these two terms prove strangely difficult to define. The developing embryo, however, reproducibly performs this task; the mechanisms underlying this process have intrigued embryologists for decades [1]. The human body shows a clear left-right (L-R) asymmetry in the placement and patterning of the internal organs and associated vasculature: the heart apex, stomach and spleen lie to the left and the liver to the right. The normal pattern of sidedness (situs), is called 'situs solitus', whereas a mirror-symmetric inversion of sidedness is called 'situs inversus'. Situs solitus is seen in almost everyone, so it is clear that the determination of situs is controlled by a robust developmental mechanism. Moreover, organ asymmetry is strongly conserved throughout the vertebrate lineage, arguing that it is of ancient origin and has been evolutionarily conserved. Defects in situs determination are rare, but when they do occur they are particularly associated with congenital heart disease (CHD) [2]. Indeed, it has been argued that very minor situs defects may manifest solely as cardiac defects, suggesting that the heart is more sensitive to situs defects than the other organs [3,4]. Strong associations are also seen between L-R defects and many ciliopathies (diseases resulting from defective cilia); this association is due to a requirement for both motile and immotile cilia in L-R determination [5,6]. L-R patterning defects also occur at a significant frequency in patients with extrahepatic biliary atresia [7]. The resultant defects in bile-duct function mean that most of these patients require a liver transplant during infancy; the mechanism underlying this association remains unknown, although simple geometric considerations may come into play.

## The role of the node in establishing L-R asymmetry in early mammalian development

**Figure 1:**
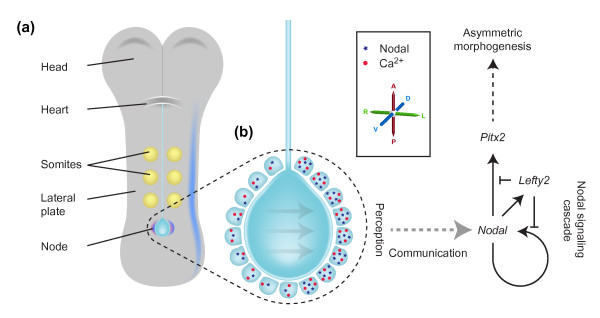
**The left-right asymmetry pathway**. **(a) **An 8.25-day mouse embryo showing asymmetric *Nodal *expression (in blue) at the node as well as in the left, but not the right, lateral plate, and asymmetric *Cerl2 *expression in purple. **(b) **A higher magnification of the node. The central pit of the node contains motile cilia (not shown) that drive a leftwards nodal flow (indicated by arrows). Surrounding the node are the node crown cells; these express Nodal (represented by blue dots) in an asymmetric fashion, with expression stronger on the left than on the right. An asymmetric calcium signal (represented by red stars) is also stronger on the left than the right. Downstream of nodal flow and asymmetric gene expression at the node, a 'leftness' signal is communicated several cell diameters to the left lateral plate mesoderm (LPM). Here it activates the Nodal signaling cascade, ultimately resulting in left-side-specific *Pitx2 *expression and asymmetric morphogenesis. The primary axes are shown: anterior-posterior (A-P); dorsal-ventral (D-V); left-right (L-R).

In the mouse embryo, the L-R axis is established at approximately 8.25 days of development. The 8.25-day embryo is relatively simple; an obvious head and heart lie at the anterior end and a midline containing the notochord runs down the middle, with the pit shaped structure known as the node at its posterior end (Figure 1). Immediately to either side of the midline sits the paraxial mesoderm, containing the somites (from which trunk muscle and skeletal tissue develop). This is flanked by the lateral plate mesoderm (LPM), a lineage that will in later development contribute to asymmetric organ structure. Overlying the mesodermal tissue is a thin layer of endoderm that gives rise to the gut. As revealed in the papers to be discussed here, the endoderm also plays a previously unrecognized part in L-R patterning.

Over the past 15 years, a general model of the establishment of L-R asymmetry has emerged (Figure 1). The first indication that bilateral symmetry of the embryo has been broken is the L-R asymmetric expression of certain genes in regions flanking the node as well as more laterally, in the LPM. Upstream of asymmetric gene expression, the rotation of motile cilia within the node (or the equivalent structure in other vertebrates) causes a leftward flow of fluid, called 'nodal flow' [8-11]. In the mouse, cilia project from the ventral surface of the node; these cilia are polarized with respect to the anterior-posterior axis and by rotating in a clockwise direction, drive nodal flow leftwards [12]. Nodal flow has been shown to be both necessary and sufficient to define the left side of the mouse embryo [13,14]. The high incidence of L-R patterning defects in humans with immotile cilia suggests that the same is true in humans [15]; approximately 50% of patients with immotile or abnormally motile cilia exhibit situs inversus [16]. Downstream of nodal flow, asymmetric Ca^2+ ^signaling is seen at the edges of the node, with stronger signaling on the left side than on the right [17]. In the interests of simplicity, this article will focus on our understanding of the determination of L-R asymmetry in the mouse, and will address two questions: how is L-R asymmetry established at the mouse node; and how is that asymmetry subsequently transferred over several cell diameters to the LPM.

## Establishing and maintaining asymmetry: the Nodal signaling cascade

Downstream of the initial breaking of symmetry at the node, the Nodal signaling cascade is activated in the left, but not the right, LPM (Figure 1). Nodal, a member of the transforming growth factor-beta (TGF-beta) signaling family of intercellular signaling proteins, functions as a dimer. Importantly, only those cells in the left and right LPM are competent to respond to Nodal signaling. In the left LPM, Nodal signaling induces expression of the *Nodal *gene itself, the *Lefty2 *gene, which encodes an antagonist of Nodal signaling, and the *Pitx2 *gene, which encodes a transcription factor that acts downstream of Nodal. Lefty2 is also a TGF-beta family member, but unlike Nodal it functions as a monomer, and diffuses faster and further than Nodal [18-20]. Once *Nodal *is expressed in the left LPM, the resultant production of Lefty2 suppresses the Nodal cascade in the right LPM, helping to lock in asymmetry [21]. *Nodal *and *Lefty2 *are expressed for only 6 to 8 hours. In contrast, once activated, *Pitx2 *remains asymmetrically expressed in the left LPM for the next two days, so that Pitx2 protein is present in the left LPM during organogenesis [22-24]. This has led to the proposition that Pitx2 is the ultimate effector of leftness [25]. While this is not absolutely the case, asymmetry of *Pitx2 *expression does underlie asymmetry of many organs [26,27].

## Detecting flow in the node: the three hypotheses

**Figure 2:**
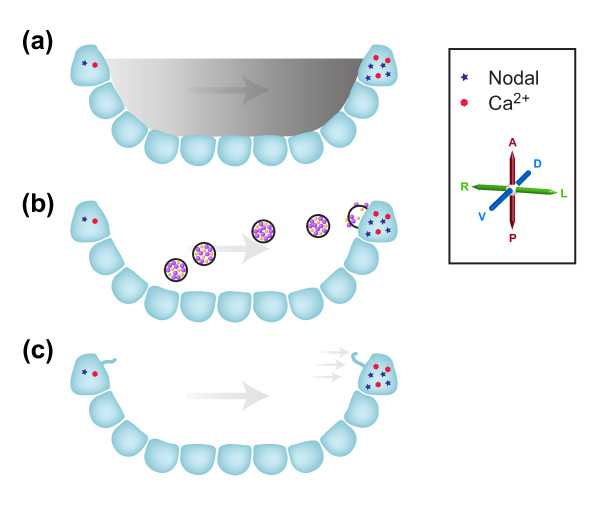
**Three models of how flow breaks symmetry at the node**. The node is represented in section, with the axes rotated by 90° from Figure 1; the axes are marked. **(a) **The morphogen hypothesis posits that a morphogen produced within the node becomes asymmetrically localized between left and right in response to flow (represented by the gray gradient). The resulting stronger left-sided signal is detected, thereby breaking symmetry. **(b) **The nodal vesicular parcel (NVP) hypothesis contends that morphogen-containing vesicles are carried leftwards by nodal flow, breaking in contact with cilia on the left side of the node. This delivers the morphogens within the NVPs asymmetrically, resulting in enriched morphogen signaling on the left-hand side of the node, thereby breaking symmetry. **(c) **The two-cilia hypothesis argues that flow itself is detected on the left side of the node by cilia-localized polycystic kidney disease (polycystin or PKD) family molecules, releasing a left-sided Ca^2+ ^signal, thereby breaking symmetry. Single cilia of crown cells on the left and right sides of the node are represented, the one on the left becoming deformed in response to nodal flow.

The mechanisms by which nodal flow is 'perceived' by the embryo remain the subject of debate, with three main hypotheses currently in contention (Figure 2). The 'morphogen hypothesis' argues that a short-lived molecule becomes enriched on the left side of the node in response to nodal flow and that this higher concentration on the left is detected, leading to a L-R asymmetric signal [8]. Both computational and experimental investigation argue that such asymmetric enrichment is plausible for molecules between 15 and 50 kDa in size, although the nature of the morphogen and the receptors are unknown [28,29]. A second hypothesis, the 'nodal vesicular parcel (NVP) hypothesis' posits the presence of membrane-bounded vesicles that are carried leftwards, breaking in a cilia-dependent fashion on the left side of the node where they deliver a cargo of morphogens [30]. Although very appealing, elements of this hypothesis (such as the mechanism of NVP breaking) clearly need to be modified [31]. Finally, the 'two-cilia hypothesis' argues that immotile sensory cilia detect flow directly on the left- but not the right-hand side of the node [17]. This model was predicated on the known function of the polycystic kidney disease 1 (*Pkd1*) and polycystic kidney disease 2 (*Pkd2*) genes in the kidney - the proteins encoded by these genes form a complex that detects urine flow and gives rise to a Ca^2+ ^signal in response [32]. Although *Pkd1 *is not required for L-R determination [33], both *Pkd2 *and the *Pkd1 *homologue Pkd1-like 1 (*Pkd1l1*) are involved in L-R patterning, being needed for the embryo to respond to nodal flow [34,35]. However, whether nodal flow pushing outwards on the left-hand side of the node can truly be differentiated from the pull exerted by nodal flow on the right-hand side of the node, a requirement of the two-cilia model, has been questioned [36]. For all these models, the outcome is an asymmetric (L greater than R) Ca^2+ ^signal at the node. Currently, only the two-cilia hypothesis (and the known function of Pkd1l1/Pkd2) provides a mechanism to explain how this signal might be generated [17].

## Investigating ciliary function

A recent study from Hamada and colleagues (Shinohara *et al*. [37]) utilizes a mixture of genetics, biophysics and imaging to examine the establishment of L-R asymmetry at the node. The authors make the striking finding that just two rotating cilia are sufficient to break L-R symmetry. In previous studies, nodal flow has been examined by following the movement of small numbers of particles across the node, allowing overall directionality and speed of flow to be assessed. For this study Shinohara *et al*. used an approach called particle image velocimetry (PIV), which they have customized for nodal-flow analysis [38]. Utilizing a high density of fluorescent beads within a living node and high-speed confocal imaging of a single optical plane, they followed small variations in particle position over many frames, building up a vector map of flow and forces across the entire node. At a local level this provides far more information than the particle-tracking approaches used up to now, and it seems likely that PIV will become a new standard for the field. With PIV analysis Shinohara *et al*. confirm that the early node (8.0 day embryo) has a weak leftward flow, which they demonstrate to be present at the time when the first asymmetric gene expression becomes apparent at the node: asymmetry of Cerberus-like 2 (*Cerl2*; also known as *Dand5*), an antagonist of Nodal signaling expressed more strongly on the right than the left side of the node. However, asymmetry of the Nodal cascade in the LPM occurs slightly later, once a stronger, more robust, leftwards nodal flow is acting at around the two- to three-somite stage (8.25-day embryo). Using a non-toxic viscous solution (methylcellulose), Shinohara *et al*. slowed, and even stopped, nodal flow. This allowed them to demonstrate that only a weak flow and/or a small temporal window of flow is required to break L-R symmetry and drive asymmetric gene expression at the node (*Cerl2*) and in the left LPM (the Nodal cascade).

In order to investigate the role of altered nodal flow in greater detail, Shinohara *et al*. sought genetic approaches to perturb it. The *Rfx3 *locus encodes a transcription factor required for normal ciliogenesis in the node; mutation of this gene leads to a massive reduction in nodal cilia number and to embryos demonstrating overt L-R patterning defects [39]. Loss of the *Dpcd *(deleted in primary ciliary dyskinesia) locus similarly results in L-R patterning defects [40]. Both loci show incomplete penetrance, which led Shinohara *et al*. to examine *Rfx3-*mutant embryos in greater detail [37]. Analysis of nodal cilia motility by light microscopy revealed that a few rotating cilia were present in these nodes. By maintaining the embryos in culture, the authors were able to image nodal cilia motility and correlate it with subsequent asymmetric gene expression. They found mildly affected embryos to have four or five rotating cilia, accompanied by normal L-R asymmetric gene expression. In contrast, severely affected embryos had at most a single rotating cilium and showed symmetrical *Cerl2 *expression at the node and complete absence of LPM Nodal expression. Further analysis revealed that only two rotating cilia were required to establish normal sidedness. In embryos with three or more rotating cilia, the addition of methylcellulose to reduce flow resulted in loss of sidedness, underlining the role of the remaining level of flow in situs determination. Finally, the position of the rotating cilia within the node (whether they were near the left or right side) was addressed, and strikingly, it emerged that their position within the node bore no relationship to their ability to determine situs: something that might have implications for all three models. Intriguingly, there also seems to be a reduction in the number of immotile cilia at the periphery of the node in these embryos, although this clearly does not affect their ability to detect flow.

It is striking that the findings of Shinohara *et al*. [37] argue for only a few motile cilia being required to establish L-R asymmetry, when perhaps 200 such cilia are present within a wild-type node. This raises the question of whether this apparent excess of cilia is truly required, or whether it is an evolutionary aberration or hangover. Unused function tends to be lost when evolutionary selection pressure is removed, as in the case of eye and pigment loss in cave-dwelling animals [41]. Three obvious possible explanations present themselves. First, the presence of higher numbers of motile cilia and prolonged flow could have subtle effects on L-R determination that are not being assessed in these studies, possibly influencing the precise timing or extent of asymmetric gene expression, or some other unknown event downstream of nodal flow. The final outcome of these events on the anatomy and physiology of the adult mouse is what is being selected. Second, the presence of many motile cilia might add robustness to the symmetry-breaking event such that deleterious outcomes (affecting cardiac patterning, for example) become extremely rare. Third, the system may not currently be under selection. In this case we might expect a loss of function to be occurring, and perhaps for variation to be evident between different strains and species of mice. Studies in other types of organism and the production of adult mice that have developed from embryos with small numbers of motile cilia may be able to shed some light on this.

Shinohara *et al*. do not directly address the question of what mechanism might underlie flow sensing in the node. The weak flow produced by two rotating cilia would primarily change morphogen concentrations only very locally, and the authors surmise that this would slow down, but not necessarily destroy, a morphogen-based mechanism. The impact of weak flow on mechanosensation (in the two-cilia model) would also be noticeable, although the authors argue how it might be possible for forces created by these cilia to be directly transduced (almost instantaneously) across the node. On the basis of developmental timing, they argue that the two-cilia model is more likely to be true.

In a subsequent study, Hamada and colleagues (Yoshiba *et al*. [42]) have addressed aspects of the mechanism downstream of nodal flow. They have investigated the role of cilia and Pkd2 in the crown cells that surround the edge of the node (Figure 1), cells that contain primarily immotile cilia. As a result of the speed at which the early embryo grows and develops, conditional gene deletion in the node is technically challenging, and it is difficult to establish whether all protein has been lost from a cell. The authors have elegantly overcome such worries by analyzing null-mutant embryos into which regionalized gene expression has been reintroduced by transgenesis. In this way they reveal that expression of *Pkd2 *(a gene normally broadly expressed in the early embryo) is required solely in the node crown cells for normal L-R patterning to occur; expression driven specifically in the remainder of the node did not rescue L-R patterning. They then showed that Pkd2 protein must be localized to cilia for it to function in L-R patterning. Finally, utilizing the *Kif3a*-null mutant (*Kif3a *encodes a motor protein required for cilia formation), Yoshiba *et al*. created embryos in which cilia were present only in the node crown cells. By then applying an artificial flow across the node, they were able to activate the normal downstream L-R pathway in these mice. This led them to propose a model in which flow is detected through cilia-localized Pkd2 protein in node crown cells, which in turn leads to repression of *Cerl2 *on the left side of the node. While these data fit well with the two-cilia hypothesis, the mechanism by which flow or a morphogen is detected remains unaddressed. Clearly, Pkd1l1 must be a prime candidate for performing this function, although it remains to be established whether Pkd1l1 responds to a morphogen or to flow [34,43].

## Transferring asymmetry from the node: intra- or extracellular communication?

**Figure 3:**
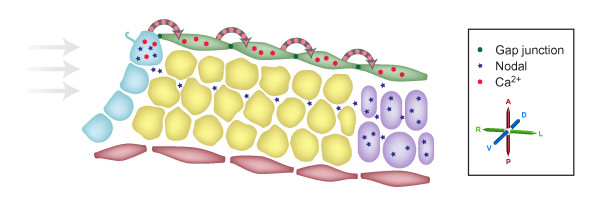
**Two models for communicating signals from the node to the left lateral plate**. A cartoon representation (not to scale) of a section through the left side of the embryo, including the left side of the node (as represented in Figure 2) and tissues lateral to the node. The cells of the node are shown in blue, the endoderm in green, the ectoderm in red, paraxial mesoderm in yellow and lateral plate mesoderm in purple. Viotti and colleagues' [49] and Saund and colleagues' work [51] argues that calcium signaling, via gap junctions, carries signals from the node leftwards through the endoderm. Oki and colleagues' analyses [48] argue that Nodal protein itself travels leftwards through an extracellular, but intraembryonic, route and directly activates the *Nodal *locus in the lateral plate.

How asymmetric information moves from the node out to the LPM is also the subject of competing hypotheses (Figure 3). When Brueckner and colleagues [17] originally described the generation of asymmetric Ca^2+ ^at the node, they commented that (at least on occasions) the asymmetric signal spread as far as the lateral plate. This clearly provides one possible mechanism by which asymmetric information might travel out from the node - by Ca^2+ ^moving intracellularly from cell to cell. In contrast, Hamada and colleagues built on two other facts: that *Nodal *expression at the node is required for *Nodal *activation in the LPM [44,45]; and the recognized ability of Nodal to activate its own expression [46,47]. They proposed that asymmetrically distributed extracellular Nodal protein at the node is transported more readily leftwards, through the extracellular matrix [48]. Two recent papers provide significant advances in our understanding of the mechanisms of these processes.

In one paper, Hadjantonakis and colleagues (Viotti *et al*. [49]) report that *Sox17*-null embryos exhibit defective L-R patterning; *Sox17 *encodes an Sry-box containing protein that is required for normal definitive endoderm formation [50]. These embryos do not express the Nodal cascade in either the left or the right LPM but, significantly, retain asymmetric gene expression at the node. This clearly suggests that communication between the node and the LPM requires the definitive endoderm, a result reminiscent of the observations of McGrath *et al*. [17]. Together, these data suggested that calcium-induced calcium release was signaling between cells in the definitive endoderm; such signals are known to travel via gap junctions. By surveying gap-junction protein expression in normal embryos, Viotti *et al*. found that the core gap-junction protein connexin 43 (also known as *Gja1*) was expressed in the definitive endoderm. However, it proved to be absent from the endoderm of *Sox17 *mutant embryos. Loss of connexin 43 does not, of course, directly prove a loss of gap-junction function. Viotti *et al*. therefore assessed this by injecting dye into definitive endoderm cells, revealing that in wild-type embryos small, but not large, dye molecules moved between cells through gap junctions. The dyes did not cross into other cell lineages and, significantly, never moved into or crossed the midline, demonstrating that the left and right sides of the embryo are distinct and not linked by gap junctions. In the absence of such a barrier, both sides of the embryo would be activated by any signal mediated through the endoderm. When *Sox17-*null embryos were examined, the dyes did not migrate between cells, demonstrating a loss of gap-junction connections. The role of gap junctions was firmly established when pharmacological agents were used to block gap-junction function in wild-type embryos, and this reproduced the L-R patterning defects seen in the *Sox17-*null embryos. This work shows that the definitive endoderm and gap junctions are required for the transfer of L-R asymmetry signals from the node to the LPM.

While Viotti *et al*. have shown that a gap-junction-dependent Ca^2+ ^signal can travel from the node to the left LPM, they stop short of providing a mechanism by which Ca^2+ ^might activate *Nodal *expression in the LPM. In contrast, a model proposed by Oki *et al*. [48] does provide an explanation for *Nodal *activation in the LPM - Nodal moving out from the node. However, the Oki model provides no explanation of the role of asymmetric Ca^2+ ^signaling in the endoderm. It is of course tempting to combine these two models: a simple combined Viotti-Oki model might propose that calcium signaling in the endoderm influences the underlying cell matrix, which in turn affects the ability of Nodal protein to propagate from the node to the left LPM.

In a contemporaneous study, Saijoh and colleagues (Saund *et al*. [51]) have independently identified the role of *Sox17 *and the definitive endoderm in L-R patterning [51]. These authors specifically investigated the link between loss of *Sox17 *function and the proposed intra-embryonic, extracellular route for Nodal. They examined extracellular matrix proteins previously implicated in the translocation of Nodal protein, revealing a defect in a proportion of *Sox17 *mutant embryos. However, a smaller proportion of embryos exhibited such defects than exhibited abnormal L-R patterning. Saund *et al*. therefore argue that this change is not the primary cause of the L-R defects in the *Sox17 *mutants. Of course, this does not fully exclude the possibility that a combination of similar defects, including those changes that they detected, are influencing intra-embryonic extracellular transport of Nodal to the left LPM.

## Prospects and questions

Although significant advances in the understanding of L-R determination have been made, gaps still remain. Even following these most recent studies, it is evident that we do not fully understand how nodal flow leads to L-R asymmetry. Both the two-cilia and morphogen hypotheses remain entirely plausible, and both have their champions within the field. Discriminating between them is not simple, and short of the identification of the putative ligand central to the morphogen hypothesis, may remain so, as if the two-cilia hypothesis is correct, no such morphogen exists. These studies are moving into the realm of the biophysicist, and will increasingly require an understanding of equations and modeling. An appreciation of the node as a (low Reynolds number) microfluidic environment, in which inertia effectively disappears, is required. In such an environment, our 'real-world' experiences can lead us to expect outcomes that are in fact incorrect, taking us far from reality; a very accessible discussion of such environments, and life at low Reynolds number, is available in the excellent article by Purcell [52].

Is it possible that both the two-cilia and morphogen mechanisms are acting in concert, providing two signals of 'leftness' in the node? Clearly, in such a scenario these might both have an impact on *Nodal *expression, but it is also possible to envisage that they might have different targets. Indeed, *Pitx2*, the most downstream gene of the Nodal signaling cascade, does not affect gross cardiac situs [26], implying that there must be additional asymmetrically expressed loci controlling this process. The simple explanation is that such loci are directly controlled by asymmetric *Nodal *expression in the LPM; in other words, that there are additional unidentified Nodal target genes at the end of the Nodal cascade. However, arguments also exists for *Nodal*-independent asymmetric gene expression in the LPM: analysis of the *Ablim1 *locus reveals it to be asymmetrically expressed in the left but not right LPM in the absence of *Nodal *expression [53]. Moreover, both galanin (*Gal*), a neuropeptide with a role in neuronal inhibition, and *Pitx2 *retain L-R asymmetric expression in early cardiac tissue (at the anterior end of the LPM) in the absence of the Nodal co-receptor Cryptic, which is required for *Nodal *expression in the LPM [54]. Whether these loci are affected by one rather than another of the putative mechanisms remains uninvestigated. Further uncertainty remains downstream of *Pitx2*, where the target genes that facilitate asymmetric morphogenesis remain to be identified.

The presence of very early L-R asymmetry, established by the initial cleavage of the embryo, has been strongly argued in *Xenopus *[55]. However, few such suggestions have been made for the mouse. The one exception is a purely embryological study by Gardner [56], which revealed that manipulation of the early blastomeres can affect the direction of embryonic axial rotation, but not other aspects of situs. While at present there is no explanation of how this might function at a molecular level, it implies that another system of L-R determination may be acting in addition to that driven by nodal flow.

The intriguing question of how neural L-R asymmetry is established in mammals remains largely unanswered. Is it linked with, or independent of, visceral asymmetry? In zebrafish, there is asymmetric expression of the Nodal cascade in the habenular nucleus of the brain (on the left but not right side), but no such association has been detected in the mouse or suggested in humans. The human *LMO4 *locus shows asymmetric expression in the brain of 12-week-old human embryos, with stronger right-sided than left-sided expression [57]. This is, however, at a much later stage of development than the establishment of visceral asymmetry, suggesting that it may be a downstream event and/or that neural asymmetry is entirely independent of visceral asymmetry. Intriguingly, expression of the mouse *Lmo4 *locus, while also asymmetrical, appears to be random, with individual embryos demonstrating a left- or a right-sided preference in their expression [57]. Whether this reflects innate differences between mouse and human brains (and neural asymmetry) remains to be determined.

Continued study, in the mouse as well as in other organisms, will be required to unravel the nature of L-R determination and its evolution. Understanding the differences between the mechanisms by which various organisms pattern their L-R axes should reveal which elements of the process have remained constant and which have varied. Ultimately, this knowledge will provide insight into the evolution of L-R asymmetry, how processes such as nodal flow have been gained and lost in different organisms, and perhaps to our understanding the evolutionary driving forces involved.
